# Inhibition of cell cycle progression by the hydroxytyrosol–cetuximab combination yields enhanced chemotherapeutic efficacy in colon cancer cells

**DOI:** 10.18632/oncotarget.20544

**Published:** 2017-08-24

**Authors:** Erika Terzuoli, Ginevra Nannelli, Maria Frosini, Antonio Giachetti, Marina Ziche, Sandra Donnini

**Affiliations:** ^1^ Department of Life Sciences, University of Siena, 53100 Siena, Italy

**Keywords:** hydroxytyrosol, cetuximab, colon cancer, cell cycle, AIF-dependent apoptosis

## Abstract

Hydroxytyrosol (HT), a polyphenol of olive oil, downregulates epidermal growth factor (EGFR) expression and inhibits cell proliferation in colon cancer (CC) cells, with mechanisms similar to that activated by the EGFR inhibitor, cetuximab. Here, we studied whether HT treatment would enhance the cetuximab inhibitory effects on cell growth in CC cells.

HT-cetuximab combination showed greater efficacy in reducing cell growth in HT-29 and WiDr cells at concentrations 10 times lower than when used as single agents. This reduction was clearly linked to cell cycle blockade, occurring at G_2_/M phase. The cell cycle arrest in response to combination treatment is related to cyclins B, D1, and E, and cyclin-dependent kinase (CDK) 2, CDK4, and CDK6 down-regulation, and to the concomitant over-expression of CDK inhibitors p21 and p27. HT and cetuximab stimulated a caspase-independent cell death cascade, promotedtranslocation of apoptosis-inducing factor (AIF) from mitochondria to nucleus and activated the autophagy process.

Notably, normal colon cells and keratinocytes were less susceptible to combo-induced cell death and EGFR downregulation.

These results suggest a potential role of diet, containing olive oil, during cetuximab chemotherapy of colon tumor. HT may be a competent therapeutic agent in CC enhancing the effects of EGFR inhibitors.

## INTRODUCTION

Hydroxytyrosol (HT), 2-(3,4-dihydroxyphenyl)ethanol, a polyphenol found in olive oil, noted for its anti-inflammatory, antioxidant activity, has been reported to reduce proliferation of human cancer cells, particularly colon carcinoma cells [1–3], and to exert pro-apoptotic effects [4–6]. Epidemiological studies substantiated the association between olive oil consumption and cancer prevention [7, 8], and a plethora of studies highlighted the beneficial properties of olive oil polyphenols due to their protective activity on DNA damage, lipid peroxidation and ROS generation [9–11]. Recently, we described the inhibition of HT on HT-29 colon cancer cell proliferation, which leads to a reduction of tumor-mass growth in a mouse HT-29 xenograft model. We also uncovered a possible mechanism, as we found that HT markedly accelerates degradation of EGFR, by promoting phosphorylation of the docking site for Cbl, a component of the ubiquitin system [3].

These findings suggest the existence of causal relationship between the HT polyphenol and the EGFR burden, widely recognized as the main driver of colon cancer growth. To exploit this concept we designed a study to investigate the influence of HT on the anticancer efficacy of cetuximab, a monoclonal antibody against EGFR known to produce EGFR downregulation, and the ensuing tumor growth inhibition and cell apoptosis. Accordingly, we compared the effects exerted by exposure to HT and cetuximab as single agents and by the HT-cetuximab combination on HT-29 and WiDr colon cancer cells, examining functional parameters of tumor growth and clonogenic potential, as well as biochemical ones. Among the latter we analyzed: EGFR expression, cell cycle progression molecules, apoptosis and autophagy markers and genes.

The HT-cetuximab combination, containing a fraction (ca. 1/10) of the maximal effective dose of each single agent, produced a far greater anti-tumor effect, in terms of inhibition of cell proliferation and clonogenic potential. Similarly, we observed consistent changes of biochemical parameters, such as EGFR expression, cell cycle check-point proteins and apoptosis, and autophagy markers.

Moreover, normal colon cells and human keratinocytes were less susceptible to cell death and EGFR downregulation induced by the combo-treatment. Interestingly, a number of recent reports have described the enhancement exerted by other polyphenols (e.g. curcumin, resveratrol) on chemotherapeutic agents on cancer progression [12, 13].

The striking enhanced efficacy on tumor cells and reduced effects on normal cells of the compound combination mentioned above might have implication in designing chemotherapeutic regimen for advanced colon cancer patients.

## RESULTS

### Growth of colon cancer cells exposed to HT and cetuximab

First, we determined the sensitivity of HT-29 and WiDr cells towards HT (1 up to 300 μM) or cetuximab (0.01, up to 100 μg/ml) alone or in presence of epidermal growth factor (EGF) for 48 h. Cells treated with 0.1% FBS were used as basal control.

**Figure 1:**
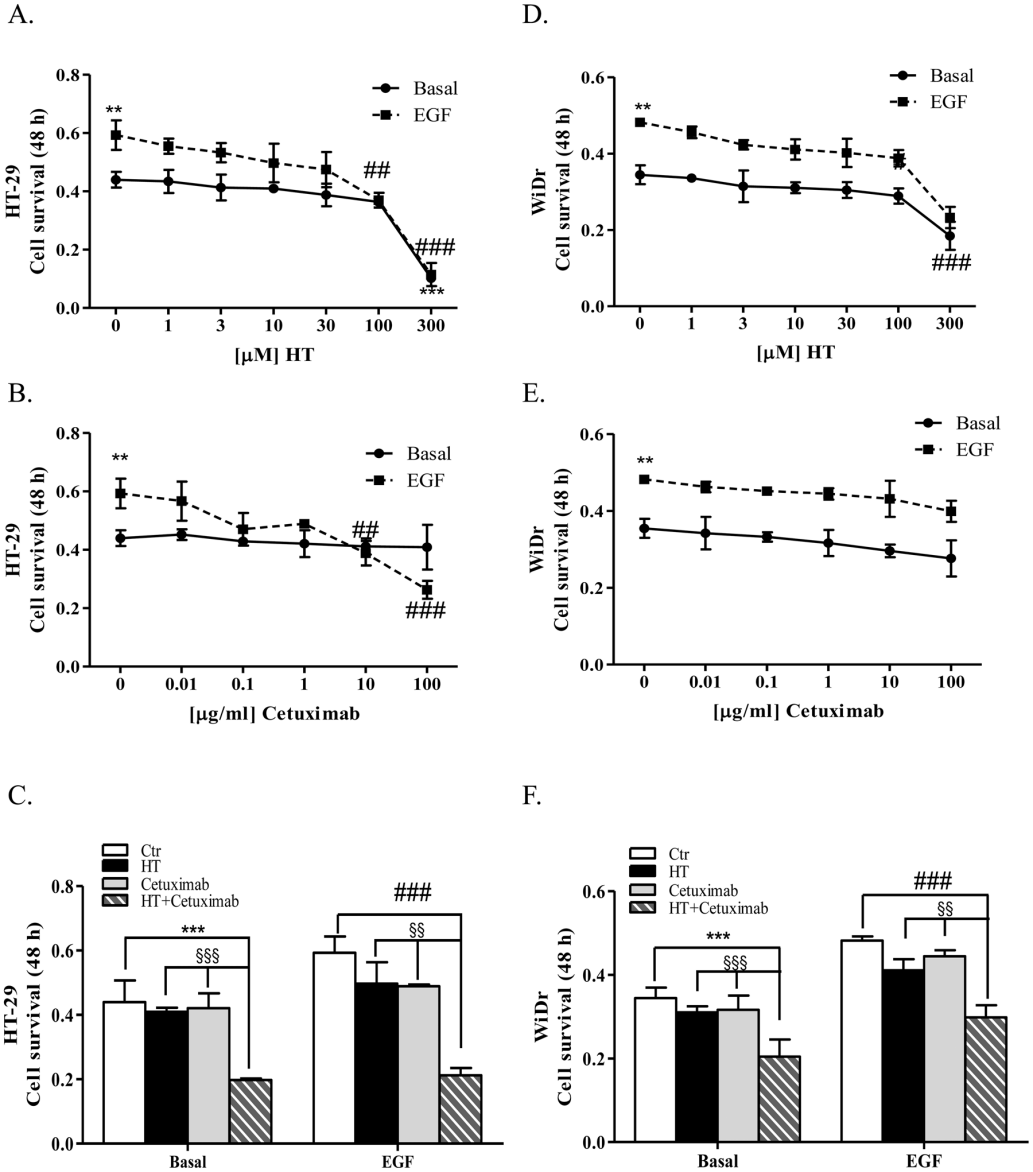
Low concentrations of HT and cetuximab reduce cell growth in colorectal cancer HT-29 **(A)**, and WiDr **(D)** cells were exposed to increasing HT concentrations in presence/absence of EGF (5 ng/ml) for 48 h. Cell viability values, reported as absorbance at 540 nm, were obtained by MTT assay. Numbers represent mean ± DS of three experiments run in triplicate ** P <0.01, *** P <0.001 vs. untreated cells. ## P <0.01, ### P <0.01 vs. EGF-treated cells. HT-29 **(B)**, and WiDr **(E)** cells were exposed to increasing cetuximab concentrations in presence/absence of EGF (5 ng/ml). Values obtained as in A. Numbers represent mean ± DS of three experiments run in triplicate. ** P <0.01 vs. untreated cells ## P <0.01, ### P <0.001 vs. EGF-treated cells. HT-29 **(C)**, and WiDr **(F)** cells were exposed to HT (10 μM) and/or cetuximab (1 μg/ml) in presence/absence of EGF (5 ng/ml) for 48 h. These concentrations were used throughout this work, unless otherwise noted. Values obtained as in A. *** P <0.001, vs. untreated cells. ### P <0.001 vs. EGF-treated cells. §§ P < 0.01. §§§ P <0.001, vs. HT or cetuximab (alone) treated cells.

The MTT assay revealed that HT reduced cell viability at the highest concentration (300 μM, P<0.001) in basal conditions, while it reduced cell proliferation in EGF-treated cells in a concentration dependent manner (Figure 1A–1D). HT IC_50_ values were 136.7 and 163.4 μM in HT-29 and WiDr cell lines, respectively. Marked differences in sensitivity to cetuximab were observed in colon cancer cells primed with EGF, as HT-29 cell growth was reduced by high cetuximab concentrations (10-100 μg/ml; IC_50_ 50.12 μM), while WiDr cells were unaffected by mAb at any concentration tested (compare Figure 1B with 1E). Cetuximab was ineffective on cell growth in control condition. High concentration of either HT (100-300 μM) or cetuximab (100 μg/ml) reduced the cell number compared to control condition, therefore useless for the scope of this work. In other experiments reported herein they were reduced to 10 μM and 1 μg/ml (HT and cetuximab, respectively) and served also for the HT-cetuximab combination (see below).

Then, we tested the combination of HT (10 μM) with cetuximab (1 μg/ml) for its effect on HT-29 and WiDr cell proliferation ability. As we noted a significant growth inhibition of the combination either in basal condition or in EGF-treated cells (Figure 1C and 1F), we conclude that HT enhances efficacy of cetuximab on tumor cells.

### HT and cetuximab reduce HT-29 and WiDr colony formation

**Figure 2:**
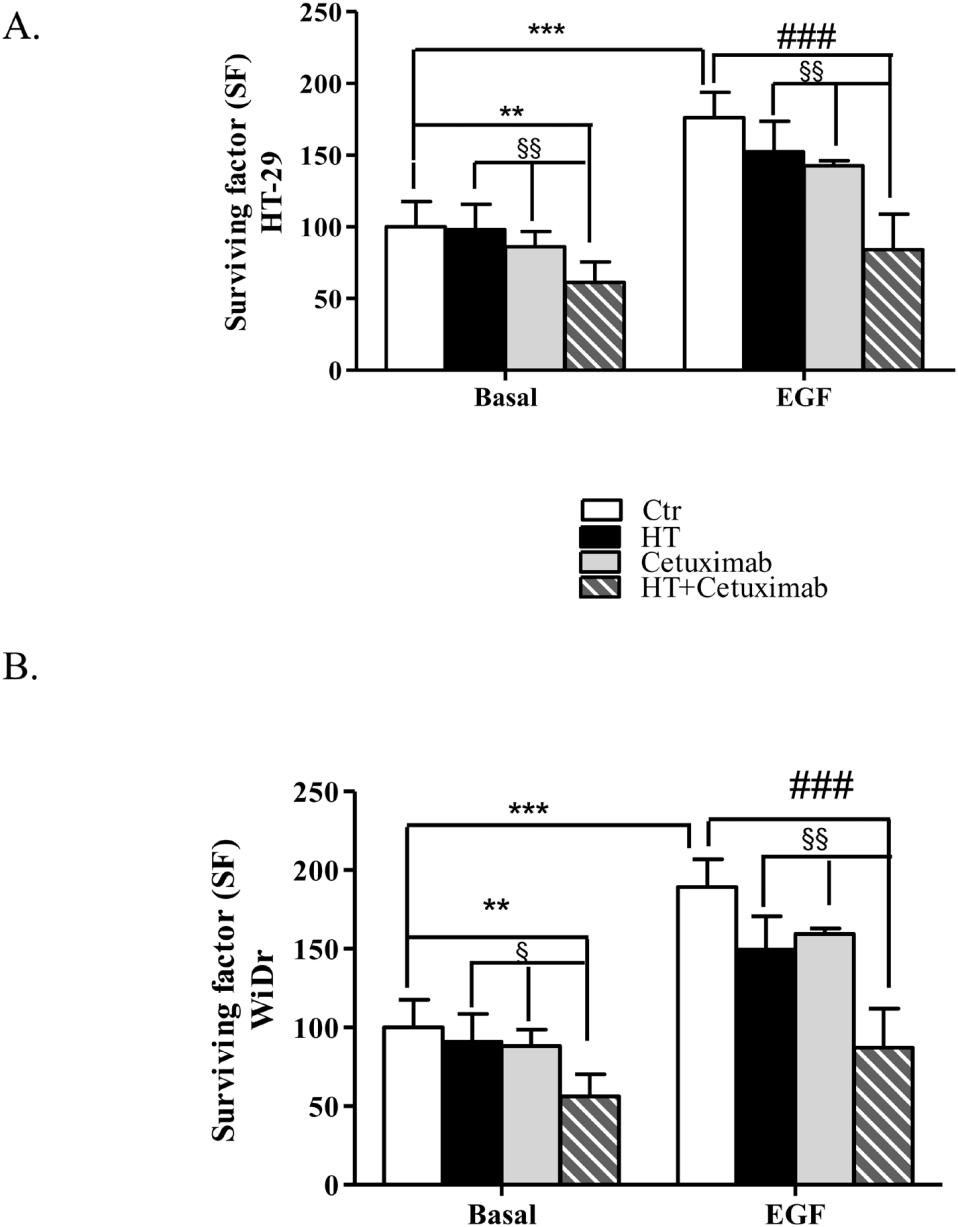
Combination of low concentrations of HT and cetuximab reduces colony formation of colorectal cancer cells Colony formation capability of HT-29 **(A)**, and WiDr **(B)** cells in response to HT (10 μM) and/or cetuximab (1μg/ml) in presence/absence of EGF (5 ng/ml). Colonies (>75 cells) with 50% efficiency were counted. Results are expressed as surviving factor (SF, see material and methods). ** P <0.01, *** P <0.001, vs. untreated cells. # P <0.05, ### P <0.001 vs. EGF-treated cells.§ P < 0.05. §§ P <0.01, vs. HT or cetuximab (alone) treated cells.

We next analyzed HT (10 μM) and cetuximab (1 μg/ml) combination on cell growth by using the functional clonogenic assay, that more closely replicates the growth characteristics of tumors *in vivo*. As shown in Figure 2A and 2B, EGF induced colony formation in both HT-29 and WiDr cells. Combination of HT and cetuximab significantly reduced colony formation both in basal and in EGF-treated cells. In both cancer cell lines, a slight reduction of clonogenic potential was observed also in presence of cetuximab alone (Figure 2A and 2B), while HT *per se* promoted a slight reduction of WiDr cells colonies only (Figure 2A).

### HT enhances cetuximab-mediated EGFR expression decline

**Figure 3:**
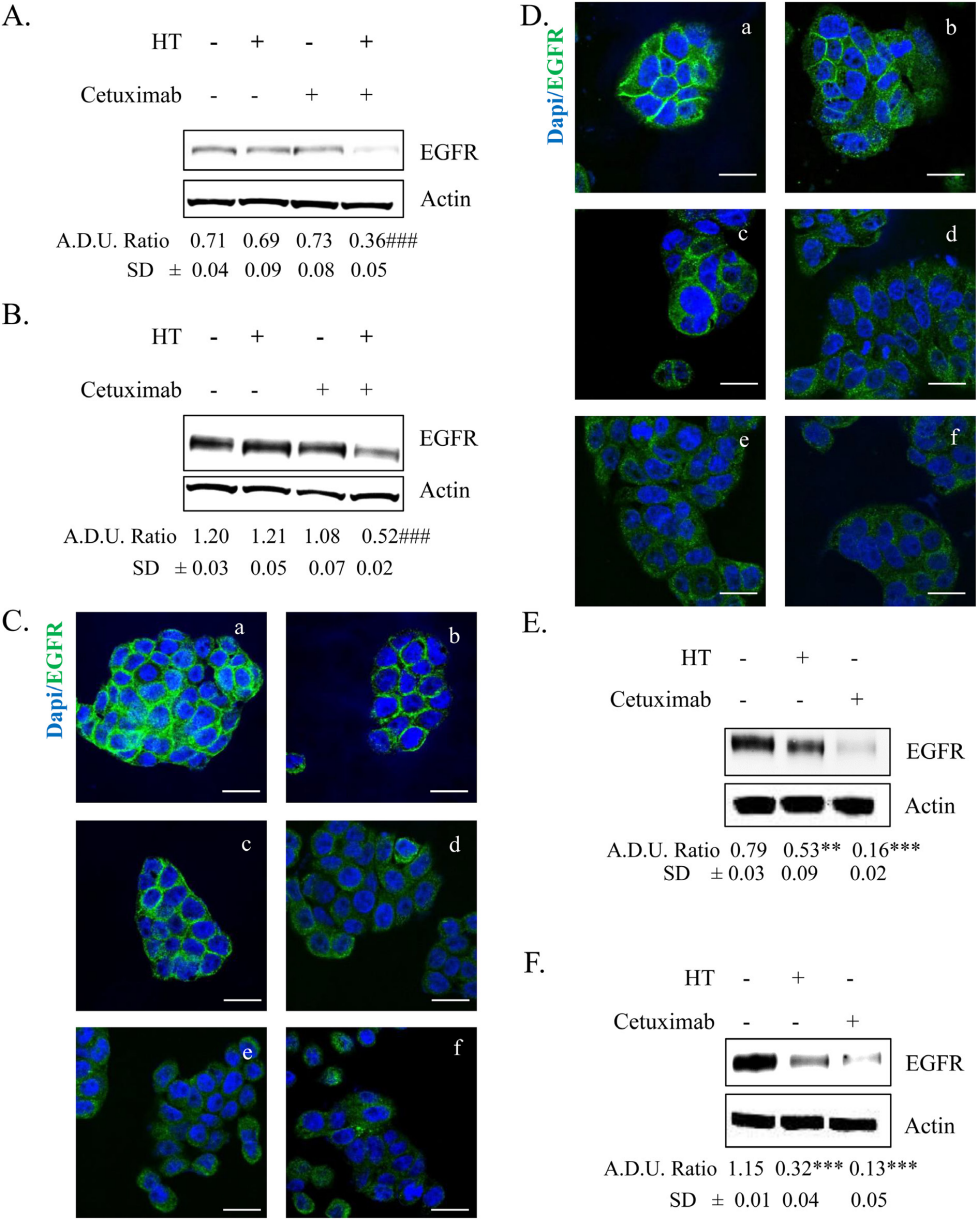
EGFR expression in colorectal cancer cells treated with HT and cetuximab alone or combined HT-29 **(A)** and WiDr **(B)** cells were exposed to low concentration of HT or cetuximab alone or in combination for 8 h and EGFR proteins was analyzed by western blot. Images of immunostaining in HT-29 **(C)** and WiDr **(D)** for EGFR (green) and DAPI (blue) in tumor cells exposed to 10 % FBS (a), cetuximab 1 μg/ml (b), HT 10 μM (c), cetuximab 1 μg/ml + HT 10 μM (d), cetuximab 10 μg/ml (e), HT 100 μM (f). Confocal images were captured with Leica SP5 confocal using 63x objective, scale bars 20 μm. HT-29 **(E)** and WiDr **(F)** cells were exposed to high concentration of HT or cetuximab for 8 h and EGFR proteins were analyzed by western blot. β-actin has been used to normalized loading (in Figures 3 and 7). Quantification (termed analysis in Figures 3 and 7; arbitrary density unit, A.D.U.) has been reported. ** P <0.01; *** P <0.001 vs. untreated cells; ### P <0.001 vs single agents.

Since reports from our and other laboratories showed that HT reduces EGFR expression [3] and cetuximab down-regulates EGFR levels in colon cancer cells [14], we investigated whether HT and cetuximab *per se* and in combination, when used at low concentrations, would affect EGFR expression in HT-29 and WiDr cells (Figure 3). Low concentration of HT and cetuximab did not reduce EGFR expression when administered *per se*, while induced a marked decline of EGFR levels when co-administered together, as assessed either by western blot or immunofluorescence (Figure 3A–3D). As expected, 8 h of treatment with high concentration of HT (100 μM) or cetuximab (10 μg/ml) reduced EGFR levels in both cell lines (Figure 3C and 3D; compare panels e and f vs panel a, and also Figure 3E and 3F) [3]. All together the data suggests that the combination of HT with cetuximab inhibits cell growth by targeting EGFR levels in colon cancer cells.

### HT and cetuximab combination arrests colon cell cycle at G2 phase

**Figure 4:**
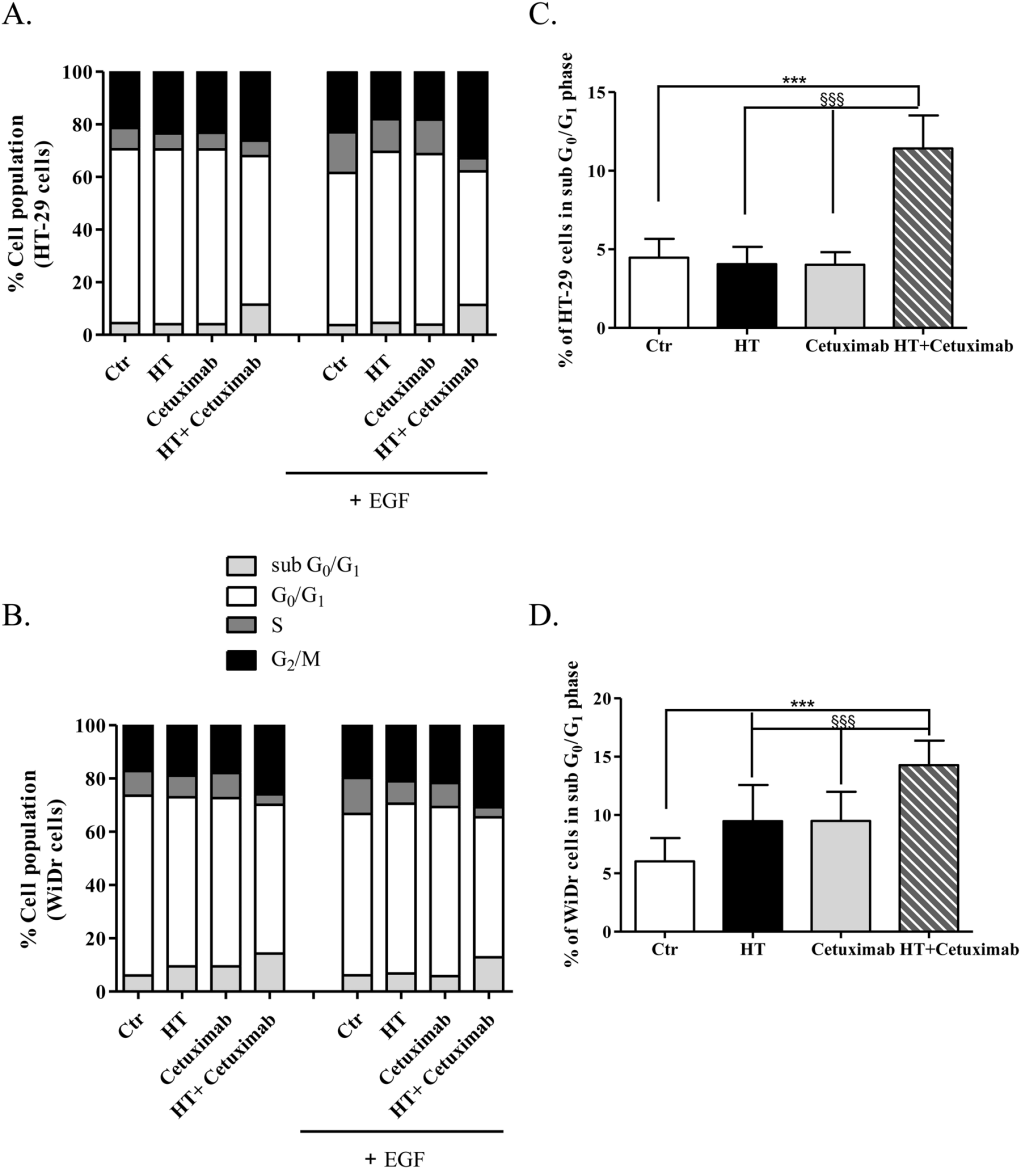
Cell cycle analysis in cancer cells treated with low concentration of HT and cetuximab combined HT-29 **(A)**, and WiDr **(B)** cells were exposed to HT or cetuximab alone or in combination in presence or absence of EGF for 48 h. The percentage of cells at each stage of the cell cycle was analyzed by flow cytometry after DNA staining with propidium iodide. Quantification of cells residing in G_0_ and G_1_ for HT-29 **(C)**, and WiDr **(D)** are reported. Percent of HT-29- (C), and WiDr-cells (D) in sub Go/G_1_ phase. *** P <0.001, vs. untreated cells. §§§ P <0.001, vs. HT or cetuximab (alone) treated cells.

In light of the cooperation observed between HT and cetuximab, we examined whether the growth inhibition might be attributed to cell cycle arrest. HT-29 and WiDr cells were treated (48 h) with EGF in the presence/absence of HT (10 μM) and cetuximab (1 μg/ml) *per se* or in combination, and labelled with propidium iodide (PI) to detect cell cycle progression by flow cytometry (Figure 4). Results showed that the treatment with HT or cetuximab did not affect the cells residing in the different phases of the cell cycle, while the combination of the two compounds caused a significant increase in the apoptotic cells represented by sub G_0_/G_1_ population (Figure 4A and 4B, and Supplementary Tables 1 and 2). Interestingly, in EGF-treated colon cancer cells, the HT-cetuximab combination challenge caused a significant increase in the sub G_0_/G_1_ population (Figure 4A and 4B, grey bars and Supplementary Tables 1 and 2), which was accompanied by accumulation of cells at G_2_/M- and by a decrease in those in S-phase (Figure 4A and 4B, black and dark grey bars, respectively, and Supplementary Tables 1 and 2). Detailed analysis revealed, in fact, that the co-treatment with HT and cetuximab induced a 3-fold and a 2-fold increase in the cells in sub G_0_/G_1_- and G_2_/M-phase, respectively, while it halved those in S phase (Supplementary Tables 1 and 2), suggesting DNA fragmentation and apoptosis process in colon cancer cells.

### HT-cetuximab combination adversely affects cell cycle checkpoint proteins in colorectal cancer cells

**Figure 5:**
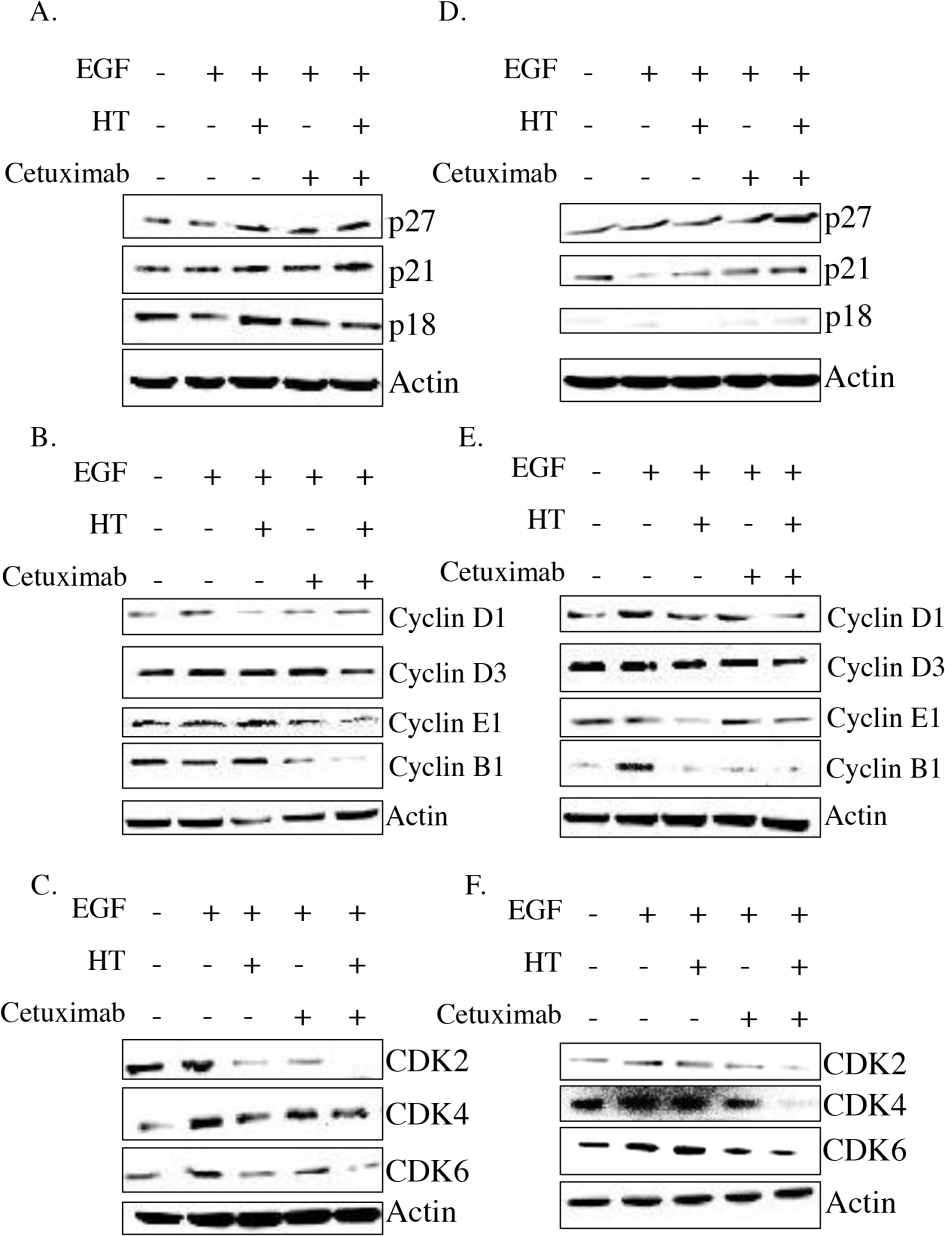
HT and cetuximab combination modulate the cell cycle checkpoint proteins in colorectal cancer cells HT-29 **(A, B, C)**, and WiDr **(D, E, F)** cells were exposed to HT or cetuximab, alone or in combination in presence of EGF for 48 h and the cell cycle checkpoint proteins were analyzed by western blot.

Analysis of the cell regulator proteins, CDKs and CDKi expression, revealed that the HT-cetuximab combo induced a significant increase in CDKi p27 and p21 expression, known to be involved in either G_1_, or G_2_, or S phase arrest, while p18 expression (barely detectable in the WiDr line) was mostly unchanged in colon cancer cells (Figure 5A and 5D Supplementary Tables 3 and 4). Furthermore, the combination reduced cyclin D1, D3, E1, CDK2, CDK4 and CDK6 expression, cell cycle regulators that mediate the transition from G_1_ to S phase, and it decreased cyclin B1, a key regulator of cells entry into mitosis (transition from G_2_ to M phase) (Figure 5B, 5C, 5E and 5F and Supplementary Tables 5–8). Thus, the HT-cetuximab combination induces G_1_/S and G_2_/M phase cell cycle arrest by reducing the expression of cell cycle regulators. (Figure 5 and Supplementary Tables 3 and 4) Of note, the concomitant downregulation of D3 and CDK6, recently reported, might disrupt the cancer-specific metabolic pathways (pentose and serine), and therefore deprive the cells of pivotal molecules such as NADPH and glutathione [15].

### HT-cetuximab combination induces cell death by activating apoptosis and autophagy processes

**Figure 6:**
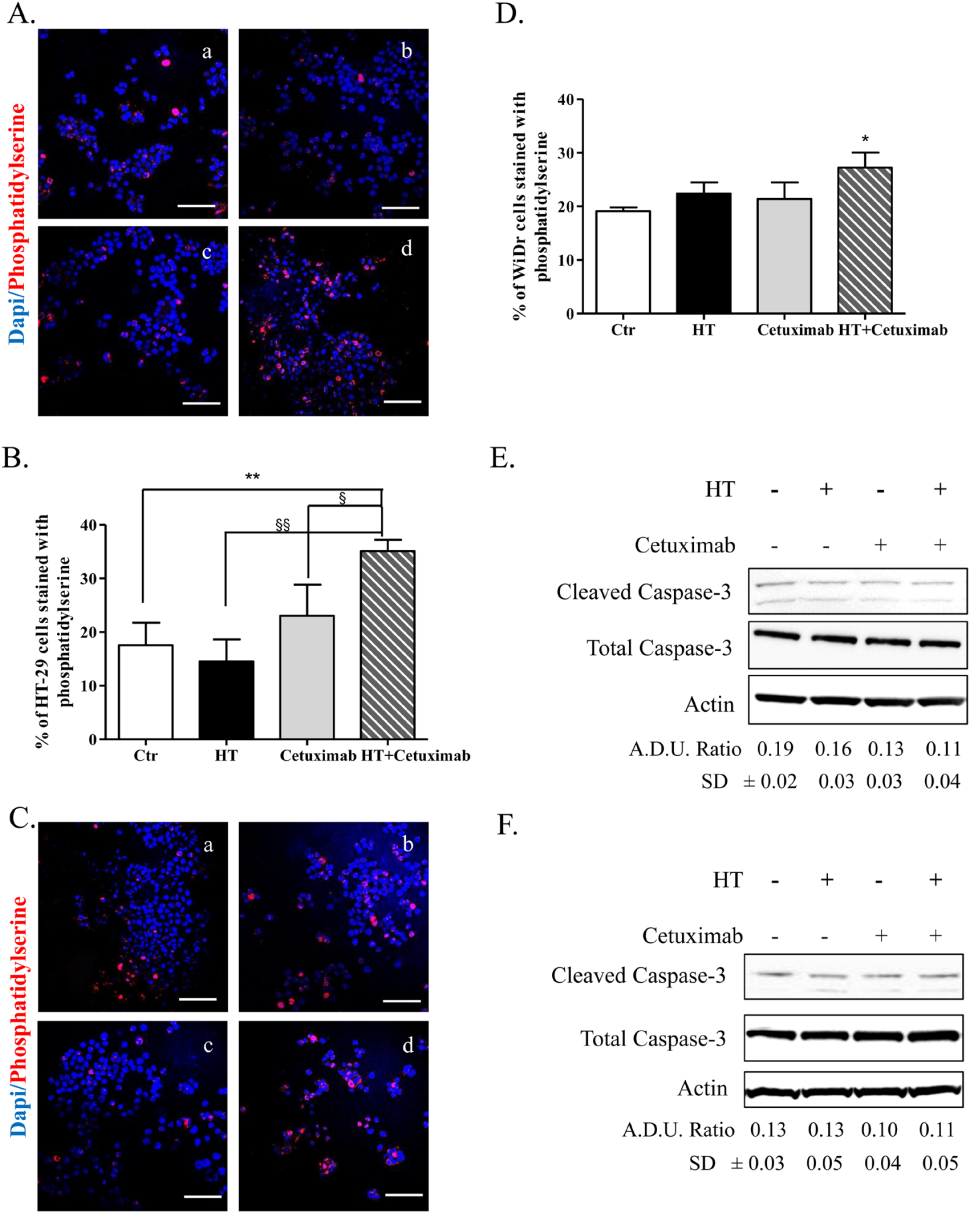
HT and cetuximab combination induces caspace3-independent apoptosis in colorectal cancer cells Phosphatidylserine (red) and DAPI (blu) exposure, assessed by immunofluorescence, in HT-29 (**A, C** for quantification) and WiDr (**B, D** for quantification) cells treated for 48 h with 10% FBS (Ctr) (a), cetuximab 1 μg/ml (b), HT HT 10 μM (c) or cetuximab 1 μg/ml+ HT 10 μM. Confocal images were captured with Leica SP5 confocal using 40 x objective, scale bars 60 μm.*P<0.05; **P <0.01 vs. untreated cells; §P<0.05; §§P<0.01 vs. HT- or cetuximab-treated cells. HT-29 **(E)**, and WiDr **(F)** cells were exposed to HT or cetuximab alone or in combination for 8 h and caspase-3 activity were analyzed by western blot. A.D.U.(cleaved caspase-3 vs. total caspase-3) has been reported.

The observed cell cycle arrest exerted by the combination provided a strong clue for studying the fate of colon cancer cells following exposure to the examined drugs. Accordingly, we investigated a number of apoptosis and autophagy markers with aim of determining the relevance of each process in the fate of cancer cells after the above treatment. Indeed, we found a robust increase of the apoptotic marker phosphatidylserine (immunostaining) solely in colon cancer cells exposed to HT-cetuximab combination, compared to control or to single agents *per se* (Figure 6A–6D). In contrast the levels of cleaved caspase-3 (Figure 6E and 6F) were unaffected. This clearly indicates that colon cancer cell death occurs in a caspase-3-independent way.

**Figure 7:**
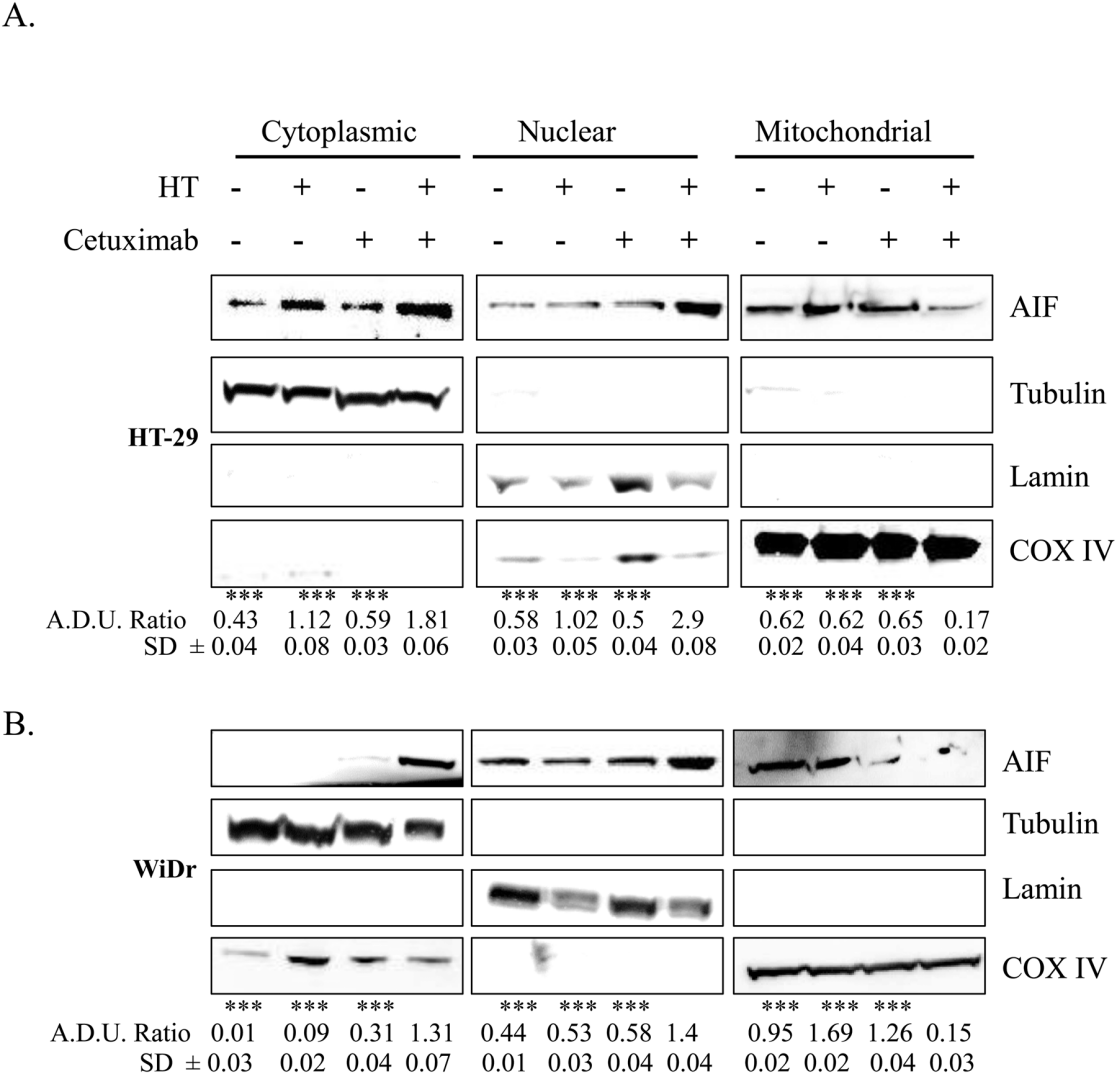
HT and cetuximab combination stimulates AIF release from the mitochondria to the cytoplasm and nuclear translocation **(A)** HT-29 (upper panel) and **(B)** WiDr (bottom panel) cells were exposed to HT or cetuximab alone or in combination for 24 h. Cytoplasmic, nuclear, and mitochondrial fractions were analyzed for AIF, tubulin (cytosolic marker), COX IV (mitochondrial marker) and lamin (nuclear marker) expression and localization Cytoplasmic AIF fraction was normalized with tubulin, nuclear fraction with lamin and mitochondrial fraction with COX IV. ***P<0.001 vs.

**Figure 8:**
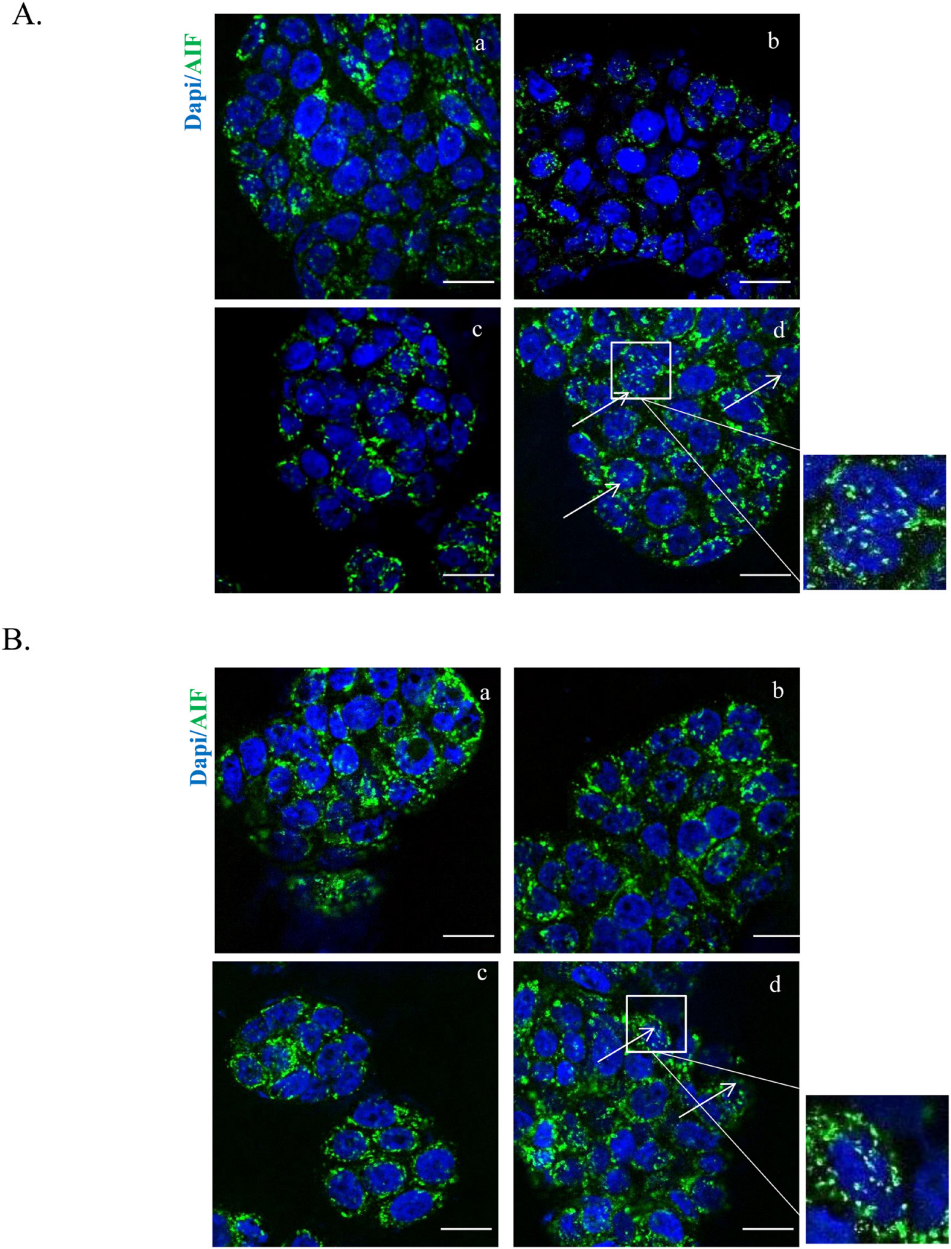
HT and cetuximab combination stimulates AIF release from the mitochondria to the cytoplasm and nuclear translocation evaluated by immunofluorescence Images of immunostaining for AIF (green) and DAPI (blue) in HT-29 **(A)** and WiDr **(B)** cells treated with 10 % FBS (a), cetuximab 1 μg/ml (b), HT 10 μM (c) or cetuximab 1 μg/ml+ HT 10 μM (d). Arrows indicate nuclei with AIF translocation. Boxed areas are shown in detail above the pictures. Confocal images were captured with Leica SP5 confocal using 63x objective, scale bars 20 μm.

Further insight was obtained by evaluating the cellular localization of AIF, an inducer of the caspase-independent pathway, which translocates to the nucleus in response to death stimuli. We found that the HT-cetuximab combination, but not the single agents *per se*, provoked AIF cytosolic and nuclear accumulation, while depriving mitochondria (Figure 7A and 7B). Immunofluorescence showed AIF an even granular distribution in cellular compartments, likely mitochondria, in control and single agent-treated cancer cells, contrasting with the striking nuclear accumulation in the combination–treated cells (Figure 8A and 8B). This suggest that the HT-cetuximab combination induces apoptosis in cancer cells via mitochondrial dysfunction, AIF translocation in nucleus and DNA fragmentation.

**Figure 9:**
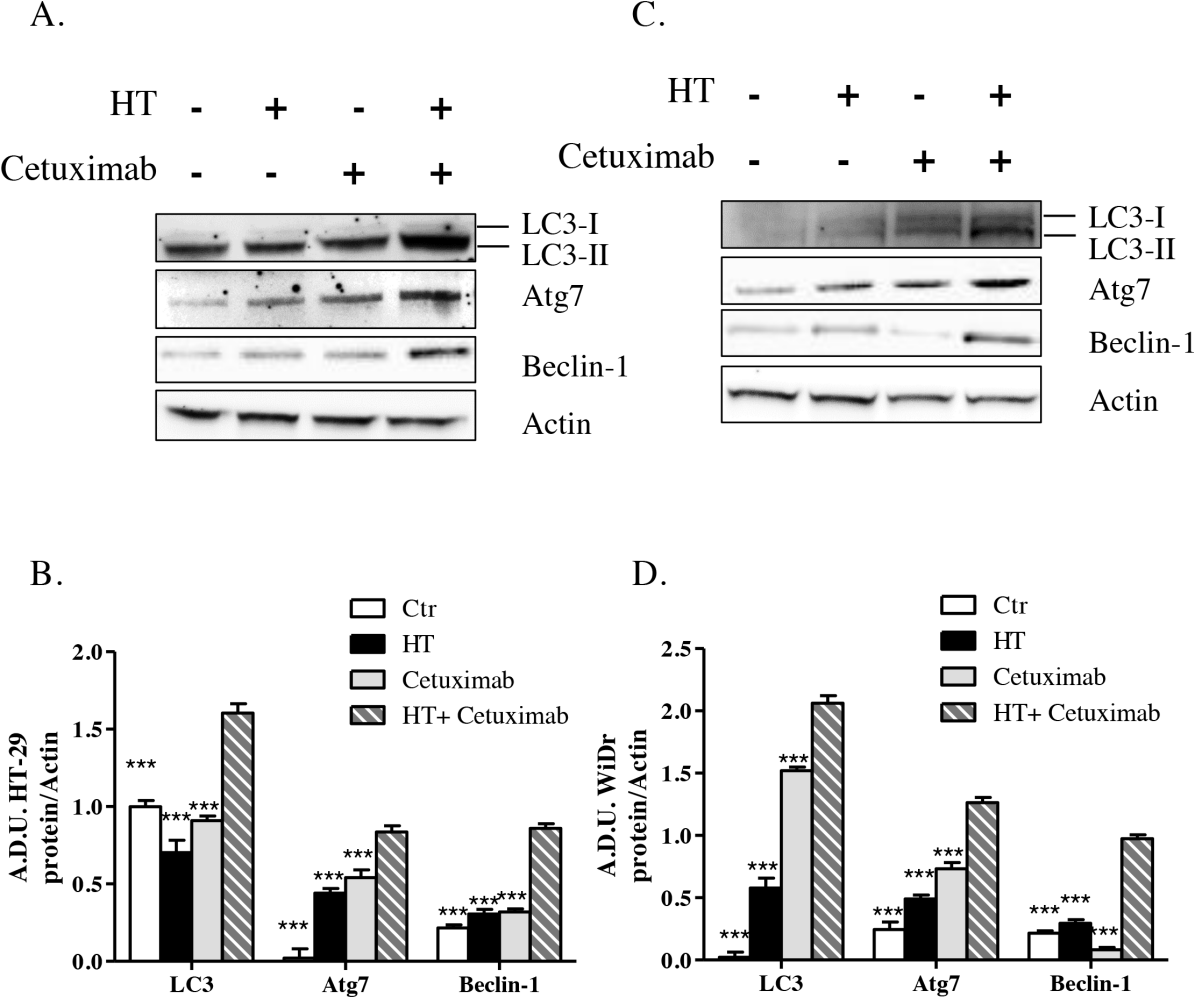
HT and cetuximab combination induces autophagy in colorectal carcinoma cells HT-29 **(A)** and WiDr **(B)** cells were exposed to HT or cetuximab alone or in combination for 24 h and the LC3, Atg7 and Beclin-1 proteins were analyzed by western blot. Quantification (**C** for HT-29 cells and **D** for WiDr cells) has been reported. *** P< 0.001 vs. HT-cetuximab combination.

Assessment of autophagy markers such as the microtubule-associated protein light chain 3 in its processed form, LC-3II, and the autophagy related gene 7, Atg7, as well as Beclin-1 levels, which were consistently overexpressed in colorectal cancer cells treated with HT-cetuximab combination relative to control and single agent treatment (Figure 9A and B), provided evidence that autophagy contributes to the anti-proliferative activities and apoptotic events described above.

### HT-cetuximab combination preserves normal colon cells and skin keratinocyte functions

**Figure 10:**
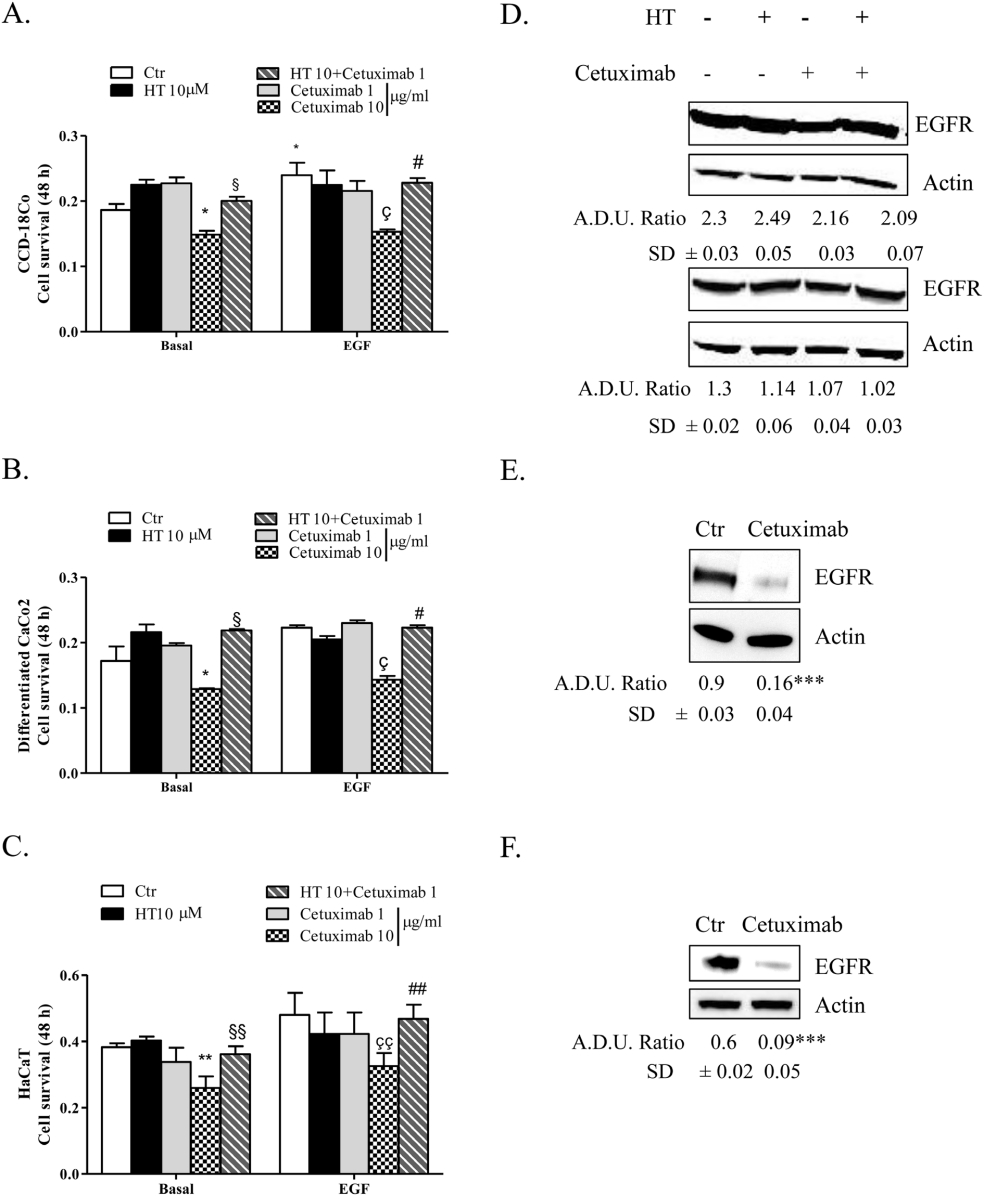
HT-cetuximab combination reduces side effects of cetuximab treatment in colorectal cancer CCD-18Co **(A)**, differentiated CaCo2 **(B)** and HaCaT **(C)** cells were exposed to the indicated concentration of HT and cetuximab in presence/absence of EGF (5 ng/ml) for 48 h. Cell survival values, reported as absorbance at 540 nm, were obtained by MTT assay. *P<0.05; **P<0.01 vs. untreated cells; §P<0.05; §§P<0.01 vs. cetuximab 10 μg/ml alone; çP<0.05, ççP<0.01 vs EGF-treated cells; #P<0.05, ##P<0.01 vs cetuximab 10 μg/ml plus EGF-treated cells. CCD-18Co (**D**, upper panel) and differentiated CaCo2 (D, bottom panel) cells were exposed to HT or cetuximab alone or in combination for 8 h and EGFR proteins was analyzed by western blot. CCD-18Co **(E)** and differentiated CaCo2 **(F)** cells were exposed to cetuximab (10 μg/ml) alone for 8 h and EGFR proteins were analyzed by western blot. β-actin has been used to normalize loading. A.D.U. has been reported. ***P<0.001 vs. untreated cells.

**Figure 11:**
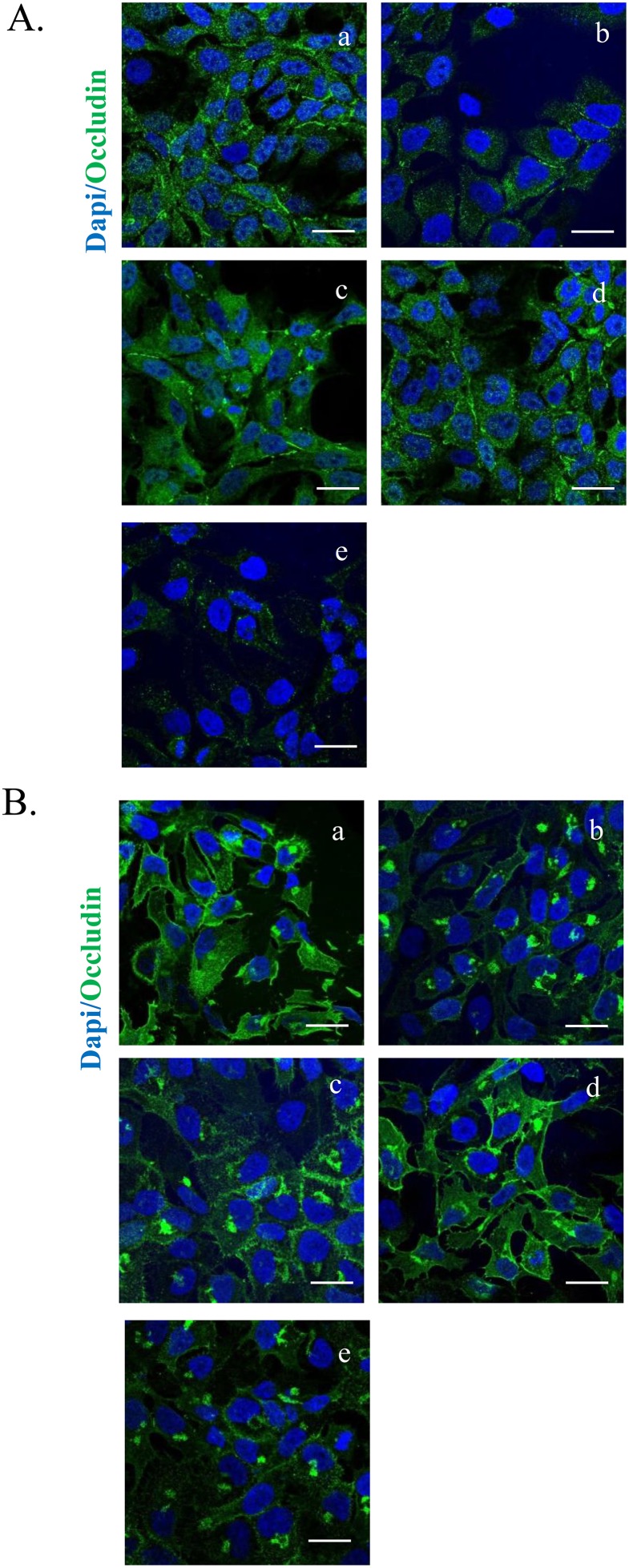
HT-cetuximab combination reduces side effects of cetuximab treatment in colorectal cancer in HaCaT cells Images of immunostaining for occludin (green) (G), claudin1 (H) and DAPI (blue) in HaCaT cells treated with 10 % FBS (a), cetuximab 1 μg/ml (b), HT 10 μM (c), cetuximab 1μg/ml + HT 10 μM (d), cetuximab 10 μg/ml (e). Confocal images were captured with Leica SP5 confocal using 63x objective, scale bars 20 μm.

We investigated whether the HT-cetuximab combination affected the normal colon cells and human keratinocytes, in terms of proliferative ability, reproducing the effects observed in colon cancer cells over single agents treatment. To this end, we used CCD-18Co (normal fibroblast colon cells), or differentiated CaCo2 cells (mimicking intestinal cells) and human keratinocytes (HaCaT) evaluating cell survival and other parameters. The HT-cetuximab combination affected neither the cell survival ability (Figure 10A–10C) nor EGFR expression levels in CCD-18Co cells or in differentiated CaCo2 cells (Figure 10D), indicating that its effect is specific for tumor cells. Further, on HaCaT cells expression of claudin-1 and Occludin proteins at the cell-to-cell contacts was only marginally affected, while at higher concentration cetuximab promoted a significant subversion of both proteins distribution on cell membranes (Figure 11A and 11B, panel e vs panel a). Altogether, these results indicate that HT-cetuximab combination exerts an antitumor effect on colorectal cancer without affecting normal colon cell function and the skin barrier of keratinocytes, possibly curtailing the dermatologic toxicity and diarrhea frequently associated with the conventional cetuximab dose regimen in cancer patients.

## DISCUSSION

Epithelial growth factor (EGF), via its receptor EGFR, elicits proliferation in many human cancers [16]. Here, we describe the effect of two molecules, cetuximab and HT, a polyphenol from olive oil, that impair the EGF/EGFR oncogenic drive, yielding a reduced proliferation of colon cancer cells (see Figure 10). Whereas cetuximab, a monoclonal antibody, occludes the EGFR binding sites, HT accelerates the EGFR degradation through ubiquitination. Although the two molecules affect tumor proliferation by disparate mechanism, it is possible that these agents might cooperate in producing an enhanced anti-tumor effect as they address the same molecular target. Evidence for cooperativity was found in functional experiments when HT and cetuximab combination (10 μM and 1 μg/ml respectively), produced greater EGFR signaling impairment than that elicited by the single agents in the colon cancer cells examined (HT-29 and WiDr). This was observed in experiments measuring either the cancer cell proliferative ability or their clonogenic propensity in response to EGF (see Figures 1 and 2). An even greater effect was detected by measuring the expression levels of EGFR, as we found a sharp decline of oncogene expression in colonic cancer cells exposed to the above combination, compared to the negligible or null effects exerted by the single agents (see Figure 3).

Analysis of cell cycle progression in cancer cells provided a rationale for the observed functional effects of the compounds examined, indicating also the underlying mechanism of action.

In fact, the HT-cetuximab combination was found to induce growth arrest at G_2_/M phase, contrasting with the negligible or null effect exerted by the single agents in this phase and in the following ones. The increase in cell number at G_2_/M phase was associated with the CDK inhibitors p21 and p27 induction [17–20], the former being known to silence the G_1_/S-promoting cyclin E/CDK2 kinase and thereby to cause a G_1_ arrest [19]. Accordingly, cyclin E and CDK2 kinases were found to be decreased by the combination. The G_1_/S checkpoint, governed by two mechanisms converging into p21, are commonly deregulated in human colon cancer [20, 21]. Moreover, the reduced cyclin B expression found in HT-cetuximab combination treated cells, signals an efficient blockade of the G_2_/M checkpoint which prevents cells from initiating mitosis in presence of DNA lesions or not fully replicated DNA [17, 18, 20, 22]. Further complexity of the cell cycle arrest arises from the recent observation showing that the down-regulation of Cyclin D3 and CDK6, as found in this work (see Figure 5 panel C trough F), has a strong impact on cancer-specific metabolic pathway (glycolysis), leading to mitochondrial dysfunction [15, 23]. In agreement, in this work we obtained evidence for mitochondrial failure following combination treatment, as we observed AIF depletion in mitochondria (see Figure 7). Although, previous studies reported that cetuximab blocks the cell cycle in G_1_ phase [24], this occurred at high concentrations (100 mg/ml). Conversely, the impairment of the EGF/EGFR system afforded by the HT and cetuximab combination appears to be related to its ability to mainly provoke a growth arrest in G_2_/M phases in colon cancer cells. Clearly, as a consequence of the ensuing DNA fragmentation observed in sub G_0_/G_1_ phase of cell cycle, we detected an activation of the apoptotic and autophagy processes in colon cancer cells exposed to the HT and cetuximab combination. Although, we found an increased phosphatidylserine externalization, the unaffected caspase-3 expression in colon cancer cells exposed to HT and cetuximab combination indicated a caspase-independent apoptosis mechanism. AIF translocation from mitochondria into nuclei, a marker of caspase-independent apoptosis, suggested that apoptosis in cancer cells is possibly related to mitochondrial dysfunction. Autophagy activation, mainly detected by a sharp rise of Beclin-1 levels, is, to a greater extent, associated with the ubiquitin system stimulation promoted by HT. However, we cannot exclude that it was associated with other effects previously reported for cetuximab [25]. The contribution of autophagy in the anti-tumoral activity of the combination needs further investigations.

**Figure 12:**
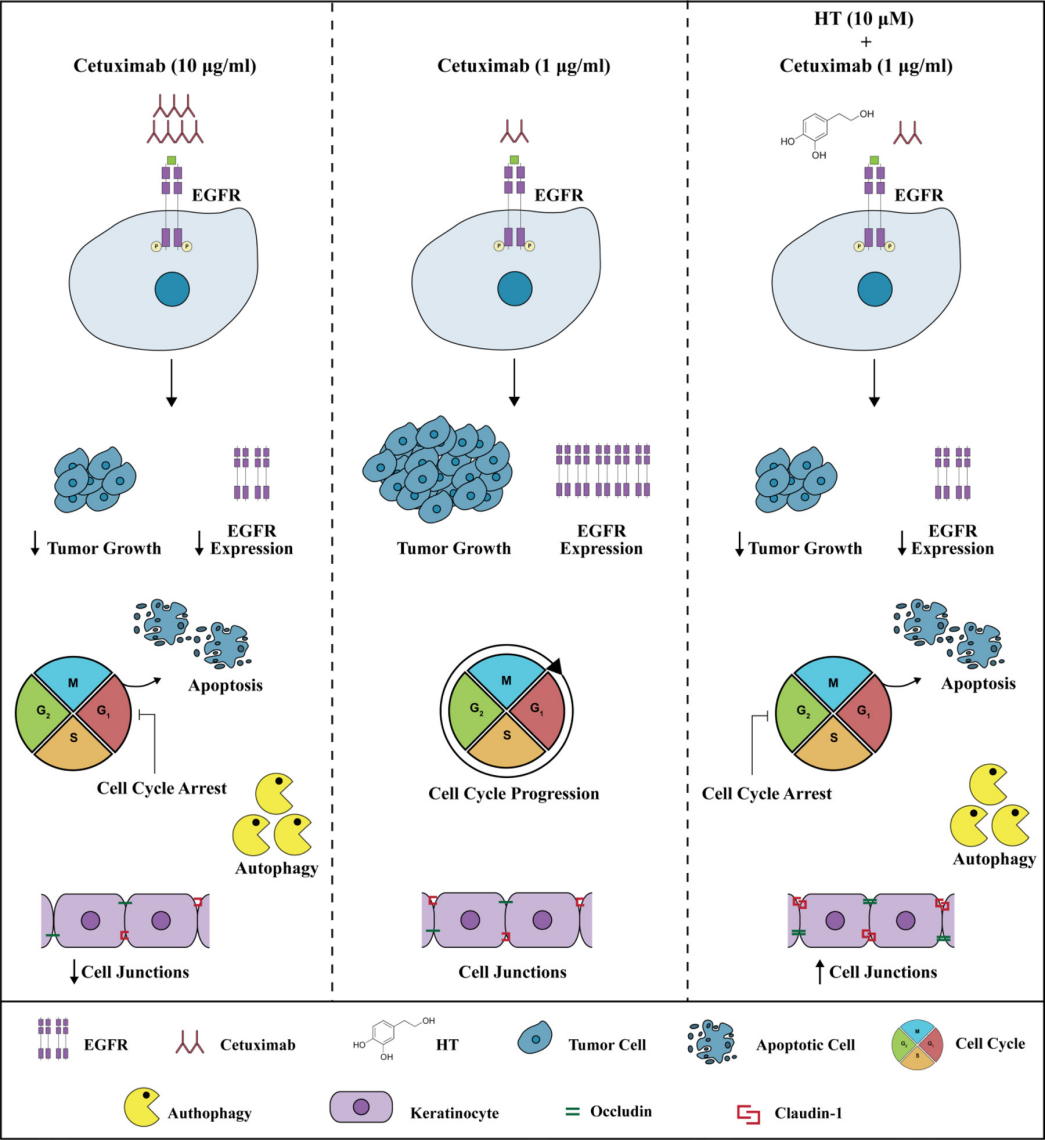
Effects of HT-Cetuximab combination on tumor and normal epithelial cells Cetuximab at 10 μg/ml, but not at 1μg/ml, blocks the cell cycle in G_1_ phase, promotes apoptosis, and autophagy in colon tumor cells, and significantly reduces cell membrane junctions in normal epithelial cells (left vs. middle panel). The combination of Cetuximab 1μg/ml with low concentration of HT (10 μM) shows cytotoxic effects on tumor cells (inhibition of cell cycle in G_2_/M phase, induction of apoptosis and autophagy), without affecting normal epithelial cells (right panel vs. middle and left panels).

Of relevance, for its clinical implications, are the findings that the HT and cetuximab combination preserved the normal colon and skin epithelial functions [26] as it failed to affect the proliferation of CCD-18Co and CaCo2 cells, as well as the membrane junctions of human keratinocytes (see Figure 12).

In sum, the polyphenol HT combined with the anti-EGFR antibody cetuximab, produces a robust inhibition of colon cancer cell growth, a remarkable effect that occurs at concentrations of each agent representing a fraction (approx. 1/10) of that producing near maximal effect as single molecules. Further, while the maximal effects of HT, as single compound, occur at concentrations borderline between physiological and pharmacological setting (100 μM), in the combination with the cetuximab, HT effects occur at physiological/nutritional setting (10 μM), suggesting that a controlled diet containing olive oil, during chemotherapy of colon tumor might enhance the effects of EGFR inhibitors.

We show evidence that the two agents cooperate in reducing the oncogenic input to colon cancer cells by two linked mechanisms. First, they accelerate EGFR turnover through ubiquitination, and curtail EGFR receptor density, enabling a reduced concentration of cetuximab to exert tumor inhibitory efficacy. Second, they provoke a cell cycle arrest at G_2_/M phase by inducing the CDK inhibitors p21 and p27 expression and the ensuing the apoptotic process. Thus, these findings may provide a rational mechanistic framework for the health benefits reported in epidemiological studies on a variety of nutritional style, particularly the well-known mediterranean diet [12, 13, 27]. Given the observed HT-induced down-shift of cetuximab potency, without compromising its antitumor efficacy, one wonders whether these findings might be translated into the cancer chemotherapy regimens in humans. This study suggests that an appropriate diet might considerably attenuate the severe side effects (e.g. skin and hematological disturbances) often associated with cetuximab and other agents with similar mechanism, which are commonly administered at maximal effective doses.

## MATERIALS AND METHODS

### Cell lines

HT-29 (passages 10-20, ATCC® HTB-38™) and WiDr (passages 5-20, ATCC® CCL-218™) human colorectal adenocarcinoma cells, CCD-18Co (passages 2–15, ATCC® CRL-1459™) human colon fibroblast cells and CaCo2 (passages 12–20, ATCCR® HTB-37™) human colorectal adenocarcinoma cells differentiated in intestinal cells as described in the next section, were obtained from the American Type Culture Collection (ATCC, LGC Standards S.r.l, Italy). HaCaT cells (passages 3-7) immortalized human keratinocytes were acquired from Voden medical (Meda, MB, Italy). All the cell lines were certified by STRA and cultured as recommended.

All the cell lines were immediately expanded after delivery (up to 6 × 10^7^ cells) and frozen down (1 × 10^6^/vial) such that all the cell lines could be restarted after a maximum of 10 passages every 3 months from a frozen vial of the same batch of cells. Control of mycoplasma was done from a frozen vial.

### Differentiation of CaCo2 cell line

CaCo2 cells were seeded in T75 flasks at a density of 6.6 × 10^4^ cells/ml and were maintained for 21 days of culture. Fresh complete growth medium was added every two days.

After the selection of 21-day cultured cells in T75 flasks CaCo2 cells were differentiated cells and used as intestinal cells [28].

### Reagents

Reagents were as follows: celLytic MT cell lysis reagent (C3228), anti-β-actin (A5441), DAPI (D9542), Fluoromount Aqueous Mounting Medium (F4680), Triton X-100 (T8787), Vybrant MTT (M5655) and crystal violet (C3886, Sigma Aldrich, Milan, Italy); HT (70604, Cayman Chemicals, VinciBiochem, Vinci, Italy); EGF (100-008, RELIAtech, VinciBiochem, Vinci, Italy), cetuximab was kindly provided by Azienda Ospedaliera Universitaria Senese, Siena, Italy.

### MTT assay

Cell proliferation was quantified by Vybrant MTT cell proliferation assay as described [29]. Briefly, cells (3 × 10^3^) were seeded in 96-multiwell plates in medium with 10 % serum for 24 h and then exposed to HT (1, 3, 10, 30, 100 or 300 μM, corresponding to the range 0.1542 – 46.26 μg/ml), cetuximab (0.01, 0.1, 1, 10 or 100 μg/ml) with/without EGF (5 ng/ml) for 48 h (with treatment of HT every 24 h). Data are reported as cell survival at 540 nm absorbance/well.

### Cell fractionation assay

To obtain cytosolic, mitochondrial and nuclear fractions, 2.5 x10^6^ cells were plated in two 10 cm diameter dishes, maintained in growth medium with 10 % FBS for 18 h and then stimulated with HT (10 μM), cetuximab (1 μg/ml) for 24 h. Cells were harvested by centrifugation at 300 × g for 5 min and re-suspended in 5 ml of 1X Buffer A to 6.6 × 10^6^ cells/ml from Abcam Cell Fractionation Kit (ab109719). Cell suspension was diluted with equal volume of Buffer B, incubated with constant mixing for 7 min at RT, and centrifuged at 5,000 × g for 1 min at 4°C. The supernatant was centrifuged at 10,000 × g for 1 min at 4°C (cytosolic fraction). The pellet was re-suspended in Buffer A, and the suspension was diluted with equal volume of Buffer C, incubated with constant mixing for 10 min at RT and centrifuged at 5,000 × g for 1 min at 4°C. The supernatant was centrifuged at 10,000 × g for 1 min at 4°C (mitochondrial fraction). The pellet was re-suspended in Buffer A (this is the nuclear fraction). All fractions were analyzed for AIF (1:1,000, Cell Signaling). Western blot was performed as described [2]. Images were digitized with the program CHEMI DOC Quantity One, blots were analyzed in triplicate by densitometry using NIH Image 1.60B5 software, and the results in arbitrary densitometric units (A.D.U.) were normalized for β-tubulin, COX IV or lamin (Sigma Aldrich).

### Immunoblot analysis

Total protein lysates were obtained using celLytic MT cell lysis reagent as described [2]. Antibodies used are as follows: anti-EGFR (4267), anti-p18 (2986), anti-p21 (2947), anti-p27 (3686), anti-cyclin D1 (2926), anti-cyclin D3 (2936), anti-CDK2 (2546), anti-CDK4 (2906), anti-CDK6 (3136), anti-cyclin B1 (4138), anti-cyclin E1 (4129), anti-AIF (5318), anti COX IV (4850), anti-LC3 I and II (12741), anti-Atg-7 (8558), anti-beclin (3495) and anti-caspase 3 (9662, Cell Signaling, Leiden, the Netherlands). Cells were stimulated with HT (10 μM), cetuximab (1 μg/ml) with/without EGF (5 ng/ml) for 8, 24 or 48 h (with administration of HT every 24 h). Images were digitalized with CHEMI DOC Quantity One program (Biorad, Hercules, CA, USA), blots were analyzed in triplicate by densitometry using NIH Image 1.60B5 software, and the arbitrary densitometric units (A.D.U.) were normalized for β-actin.

### Immunofluorescence

3x10^4^ cells were cultured on coverslips, treated with HT (10-100 μM), cetuximab (1-10 μg/ml) with/without EGF (5 ng/ml) for 8-24 or 48 h (with treatment of HT every 24 h) and then fixed in paraformaldehyde (4%, 10 min), washed in PBS and permeabilized, if necessary, with Triton X-100 (0.5% in PBS containing 0.5% BSA) and then incubated with BSA (45 min). Cells were then incubated for 16 h with anti-EGFR (1:40), anti-AIF (1:50, Cell Signaling), anti-phosphatedylserine (1:50, 05-719 Merk Millipore), anti-occludin (1:80, 71-1500 ZYMED) diluted in PBS containing 0.5% BSA. To investigate phosphatedylserine externalization on cell membranes, cells were fixed in paraformaldehyde (4%, 10 min), washed in PBS, incubated with BSA (45 min) without permeabilization, and then incubate for 16 h with anti-phosphatedylserine (1:50, 05-719 Merk Millipore) diluted in PBS containing 0.5% BSA. After incubation with the secondary antibody ALEXAFLUOR 488 anti-mouse or anti-rabbit (A11001, A11011 respectively; 1:200 1 h, Thermo Fisher), cells were washed and incubated with DAPI (1 μg/mL, 20 min), washed and the coverslips were mounted in Fluoromount Aqueous Mounting Medium.

### Cell cycle

Cell cycle distribution was analyzed using flow cytometry after propidium iodide staining. Colon cells (7 × 10^5^) were seeded in 6-multiwell plates in growth medium with 10% serum for 24 h, left overnight to allow for cell attachment, and then exposed to 0.1 % serum for 24 h and then treated with HT (10 μM), cetuximab (1 μg/ml) with/without EGF (5 ng/ml) for 48 h (with treatment of HT every 24 h). Cells were then washed three times with PBS, trypsinized and collected by centrifugation at 0.3 × g for 5 min. The cells were fixed overnight in 80% ethanol at −20°C, then washed twice with PBS and incubated with 0.5 ml of PBS containing 100 μg/ml RNase (R6148, Sigma Aldrich) and 50 μg/ml propidium iodide (P4170, Sigma Aldrich) at 37° C for 30 min. Cell cycle distribution was analyzed by measuring DNA content using a flow cytometer (BD Bioscences, Milan, Italy). Data are analyzed in triplicate by Cell Quest Pro, BD [30, 31].

### Clonogenic assay

For clonogenic assay, HT-29 and WiDr cells, were stimulated with HT (10 μM) and cetuximab (1 μg/ml) for 48 h (with treatment of HT every 24 h) with/without EGF (5 ng/ml). Following treatments, cells were plated in 60 mm culture dishes (at a density of 1,000 cells/dish) in medium containing 1% fetal calf serum, and then kept in a humidified incubator at 37° C and 5% CO_2_ for 2 weeks. Colonies (400 cells) with 50% plate efficiency were fixed and stained with 0.05% crystal violet in 10% ethanol and counted. Data are expressed as surviving factor (SF), that indicates the number of colonies that arise after treatment of cells, expressed in terms of PE (PE is the ratio of the number of colonies to the number of cells seeded) [32].

$$\text{SF}=\frac{\text{no. of colonies formed after treatment}}{\text{no. of cells seeded × PE}}$$

### Statistical analysis

Results are expressed as means ± SD or ±SEM. Statistical analysis was carried out using Student's t test, two-away ANOVA, followed by Bonferroni post-test for multiple comparison. P<0.05 was considered statistically significant.

## SUPPLEMENTARY MATERIALS TABLES



## Abbreviations

AIF: apoptosis inducing factor
CC: colon cancer
CDK: cyclin-dependent protein kinases
EGF: epidermal growth factor
EGFR: epidermal growth factor receptor
HT: hydroxytyrosol or 2-(3,4-dihydroxyphenil)ethanol
mAbs: monoclonal antibodies
PBS: phosphate buffer saline
PE: ratio of the number of colonies to the number of cells seeded
SD: standard deviation
SF: surviving factor
