# Supplementary material for: Inhibition of cell cycle progression by the hydroxytyrosol–cetuximab combination yields enhanced chemotherapeutic efficacy in colon cancer cells
Source: Oncotarget. 2017 Aug 24;8(47):83207–24. doi: 10.18632/oncotarget.20544 (PMC5669961; doi:10.18632/oncotarget.20544)
Supplement: Supplementary file 1 [file oncotarget-08-83207-s001.pdf]

Inhibition of cell cycle progression by the hydroxytyrosol–cetuximab combination yields enhanced chemotherapeutic efficacy in colon cancer cells

SUPPLEMENTARY MATERIALS

Supplementary Table 1: Cell cycle analysis in cancer cells treated with low doses of HT and cetuximab combined in Figure 4

HT-29 cells	% of cells in phase sub G_0/G_1	% of cells phase G_0/G_1	% of cells in phase S	% of cells in phase G_2/M
0.1 % FBS	4.47±1.2	66.09±5.8	8.04±1.2	21.4±3.5
HT	4.06±1.09	66.41±12	6.03±2.1	23.5±4.9
Cetuximab	4.02±0.8	66.4±8.9	6.37±2	23.2±5.6
HT + Cetuximab	11.42±2.1***	56.5±7	5.9±2.3	26.15±4.6
0.1% FBS + EGF	3.69±1.2	57.81±8.9	15.64±2.1	22.83±5.8
HT+ EGF	4.54±0.788	65.01±5.6	12.43±1.3	17.93±4.7
Cetuximab + EGF	3.91±1.1	64.83±7.8	13.02±1.1	18.22±3.4
HT + Cetuximab + EGF	11.36±2###	50.77±11	4.97±1.5###	32.9±3.5#

HT-29 cells were exposed to HT (1 µM) or cetuximab (10 µg/ml) alone or in combination in presence of EGF (25 ng/ml) for 48 h. The percentage of cells at each stage of the cell cycle was analyzed by flow cytometry after DNA staining with propidium iodide.
***P<0.001 vs. Cetuximab-treated cells. #P<0.05, ###P<0.001 vs. cetuximab + EGF-treated cells.

Supplementary Table 2: Cell cycle analysis in cancer cells treated with low doses of HT and cetuximab combined in Figure 4

WiDr cells	% of cells in phase sub G_0/G_1	% of cells in phase G_0/G_1	% of cells in phase S	% of cells in phase G_2/M
0.1 % FBS	6.03±2	67.54±3.5	9.1±1.1	17.22±3.3
HT	9.48±3.11	63.49±5.6	8.03±1.9	19±4.1
Cetuximab	9.49±2.5	63.14±8.7	9.37±2.4	18±3.2
HT + Cetuximab	14.27±2.1*	55.84±4.9	9.89±2.1	27±4.1
0.1% FBS + EGF	6.15±1.1	560.59±7.8	13.44±1.4	19.28±0.9
HT + EGF	6.77±2.7	63.79±5.6	8.43±1.5	21.01±4.2
Cetuximab + EGF	5.77±2.9	63.48±6.7	9.02±1.3	21.73±7.1
HT + Cetuximab + EGF	12.84±3.4###	52.59±8.3	3.77±1.7##	30.8±1.5##

WiDr cells were exposed to HT (1 µM) or cetuximab (10 µg/ml) alone or in combination in presence of EGF (25 ng/ml) for 48 h. The percentage of cells at each stage of the cell cycle was analyzed by flow cytometry after DNA staining with propidium iodide.

*P <0.05, vs. Cetuximab-treated cells. ##P<0.01, ###P <0.001 vs. cetuximab + EGF-treated cells.

Supplementary Table 3: Quantification of p18, p21 and p27 protein expression in HT-29 cells

A.D.U. ± SD	P27	P21	P18
**Basal**	0.30±0.03	0.43±0.02	0.83±0.05
**EGF**	0.27±0.02	0.48±0.04	0.63±0.04
**HT + EGF**	0.420±0.05##	0.51±0.04	0.84±0.03
**Cetuximab + EGF**	0.39±0.03#	0.49±0.03	0.71±0.02
**HT + Cetuximab + EGF**	0.72±0.04ççç	0.73±0.05ççç	0.61±0.01

Supplementary Table 4: Quantification of p18, p21 and p27 protein expression in WiDr cells

A.D.U. ± SD	P27	P21	P18
**Basal**	0.32±0.03	0.36±0.02	0.24±0.05
**EGF**	0.46±0.02	0.05±0.04***	0.23±0.04
**HT + EGF**	0.40±0.05	0.24±0.04#	0.12±0.09
**Cetuximab + EGF**	0.45±0.03	0.31±0.01#	0.27±0.02
**HT + Cetuximab + EGF**	0.89±0.0ççç	0.51±0.05ççç	0.32±0.01

Supplementary Table 5: Quantification of cyclin D1, D3, E1 and B1 protein expression in HT-29 cells

A.D.U. ± SD	Cyclin D1	Cyclin D3	Cyclin E1	Cyclin B1
**Basal**	0.22±0.03	0.57±0.02	0.44±0.05	0.78±0.05
**EGF**	0.37±0.02**	0.75±0.04*	0.54±0.04*	0.57±0.04*
**HT + EGF**	0.240±0.05##	0.85±0.04	0.65±0.03	0.79±0.09#
**Cetuximab + EGF**	0.23±0.03##	0.87±0.0	0.46±0.02	0.56±0.02
**HT + Cetuximab + EGF**	0.19±0.04ç	0.59±0.05çç	0.25±0.0ççç	0.07±0.01ççç

Supplementary Table 6: Quantification of cyclin D1, D3, E1 and B1 protein expression in WiDr cells

A.D.U. ± SD	Cyclin D1	Cyclin D3	Cyclin E1	Cyclin B1
**Basal**	0.43±0.03	0.94±0.02	0.60±0.05	0.08±0.05
**EGF**	0.68±0.02*	0.83±0.04	0.38±0.04*	0.58±0.04***
**HT + EGF**	0.38±0.05#	0.58±0.04#	0.28±0.09	0.05±0.09###
**Cetuximab + EGF**	0.43±0.03#	0.67±0.01#	0.38±0.02	0.09±0.02###
**HT + Cetuximab + EGF**	0.18±0.04çç	0.40±0.05çç	0.23±0.01ç	0.05±0.01

Supplementary Table 7: Quantification of CDK2,4 and 6 protein expression in HT-29 cells

A.D.U. ± SD	CDK2	CDK4	CDK6
**Basal**	0.54±0.03	0.21±0.02	0.25±0.05
**EGF**	0.50±0.02	0.53±0.04***	0.34±0.04*
**HT + EGF**	0.07±0.05###	0.29±0.04##	0.13±0.03##
**Cetuximab + EGF**	0.11±0.03###	0.39±0.03##	0.21±0.02#
**HT + Cetuximab + EGF**	0.007±0.04ççç	0.36±0.05	0.06±0.01ççç

Supplementary Table 8: Quantification of CDK2, 4 and 6 protein expression in WiDr cells

A.D.U. ± SD	CDK2	CDK4	CDK6
**Basal**	0.21±0.03	1.41±0.02	0.64±0.05
**EGF**	0.39±0.02*	1.95±0.04**	0.99±0.04*
**HT + EGF**	0.37±0.05	1.81±0.04	1.07±0.09
**Cetuximab + EGF**	0.23±0.03	1.2±0.01#	0.65±0.02#
**HT + Cetuximab + EGF**	0.07±0.04ççç	0.08±0.02çç	0.41±0.01çç

Quantification of cell cycle checkpoint proteins (see Figure 5 for images) in HT-29 cells (3, 5, 7) and WiDr cells (4, 6, 8) exposed to HT or cetuximab (alone or in combination) in presence of EGF for 48 h and analyzed by western blot. *P<0.05, **P<0.01, ***P<0.001 vs. untreated cells. #P<0.05, ##P<0.01, ###P<0.001 vs. EGF-treated cells. çP<0.05, ççP<0.01, çççP<0.001 vs. HT or cetuximab + EGF-treated cells.
